# First Report of the Gene Mutations Associated with Permethrin Resistance in Head Lice (*Pediculus humanus capitis* De Geer, 1767) from Primary School Children in Istanbul (Türkiye) and Nagarkot (Nepal)

**DOI:** 10.3390/pathogens13121116

**Published:** 2024-12-17

**Authors:** M. Burak Batır, Yeşim Yasin, Anuradha Jaiswal, Tuana Tabak, Özgür Kurt

**Affiliations:** 1 Department of Biology, Faculty of Engineering and Natural Sciences, Manisa Celal Bayar University, Manisa 45140, Türkiye; burak.batir@cbu.edu.tr; 2 Department of Public Health, Acibadem University School of Medicine, Istanbul 34752, Türkiye; yesim.yasin@acibadem.edu.tr; 3 Department of Pediatrics, BPKIHS, Tribhuwan University School of Medicine, Kathmandu 67201, Nepal; anuradhaj144@gmail.com; 4 Acibadem University School of Medicine, Istanbul 34752, Türkiye; tuanatolunay@gmail.com; 5 Department of Medical Microbiology, Acibadem University School of Medicine, Istanbul 34752, Türkiye; 6 Clinical Parasitology Study Group, European Society of Clinical Microbiology and Infectious Diseases (ESCMID), 4051 Basel, Switzerland; 7 Parasitology Study Group, Turkish Society of Microbiology, Istanbul 34093, Türkiye

**Keywords:** *Pediculus humanus capitis*, head lice, permethrin, resistance, Turkey, Nepal

## Abstract

Head lice infestation (HLI), caused by *Pediculus humanus capitis* De Geer, 1767, has long been a common global problem of school children. Permethrin is an old pyrethroid derivative that has been used commonly for its treatment, and it exerts its activity over the voltage-sensitive calcium channels (VSCC) of the lice. There has been a growing list of persistent HLI cases lately in the world among patients using permethrin, and knockdown resistance (kdr)-related point mutations on VSCC have been identified and reported from those resistant lice samples. The aim of this study was to investigate the gene mutations associated with permethrin resistance in head lice collected from primary school children in Istanbul (Türkiye) and Nagarkot (Nepal) for the first time. A total of 192 *P. h. capitis* adults were collected from school children aged 6–12 years in two cities (96 lice each). Following DNA isolation, the fragment of the VSCC a-subunit gene, which contained the possible mutation sites ((kdr-like M815I (ATG > ATT), kdr T917I (ACA > ATA), and kdr-like L920F (CTT > TTT)), was amplified in each louse by PCR, and the PCR products were sequenced and aligned, followed by frequency calculations for alleles, genotypes, and haplotypes. Using nucleic acid sequence analysis, it was revealed that M815I, T917I, or L920F mutations were present on the VSCC genes in the lice samples from both Türkiye and Nepal. In addition, genotypic analyses indicated the presence of all three mutations in the lice samples from Türkiye, while the T917I mutation was detected in none of the lice collected in Nepal. This is the first report of gene mutations associated with permethrin resistance in head lice collected from a group of primary school children in the largest city of Türkiye (Istanbul) and Nagarkot. High mutation rates were identified in the lice, especially those from Istanbul, which is concordant with our previous unpublished study, in which almost 60% of the examined lice of the school children (in the same school selected in this study) remained alive despite long-term exposure to permethrin in the laboratory. These initial results show that gene mutations associated with permethrin resistance are common in lice samples in Istanbul and Nagarkot, which may suggest the current need for the selection of new pediculicidal agents in HLI treatment.

## 1. Introduction

Head lice infestation (HLI) is a common public health problem and nuisance for humans, especially for young children in the world. It is caused by *Pediculus humanus capitis* De Geer, 1767, which is a human-specific arthropod transmitted by direct contact and associated with severe itching and discomfort in patients who are predominantly day-care and primary school students or residents of dormitory schools, prisons, or mental health institutes [1,2]. Secondary bacterial infections are not uncommon in patients, due to intense itching of the scalp, while recent reports suggest that head lice harbor and may transmit life-threatening bacteria to humans, such as *Acinetobacter baumanii*, *Rickettsia prowazekii*, and *Bartonella quintana* [1,2,3,4].

Today, almost one out of five school children in the world are infested with *P. h. capitis* [2]. Previous studies conducted on a total of 578,938 individuals in Türkiye between 1982 and 2012 showed that the frequency of HLI was within 0.3–1% [5]. In Nepal, HLI frequency was reported to be 16% and 59% in individuals aged between 0 and 39 years and homeless children, respectively, and 21% in a group of urban school children in two studies [6,7]. The prevalence rates of HLI are higher in girls in almost all countries, probably due to their longer hair [1]. Therefore, effective treatment and continuous monitoring are essential to overcome this problem, which may sometimes appear as an epidemic among children.

Permethrin is a well-known pediculicidal agent and has been commonly used in the treatment of HLI [3]. As a pyrethroid, it exerts its effects by depolarizing the voltage-sensitive sodium channels located on the cell membranes of head lice, which eventually causes the paralysis of the louse [8]. It was the mainstay of treatment of HLI worldwide for a long time, as it was an inexpensive and available agent; however, concerns have been on the rise lately about its toxicity to the scalp, especially in young children, and there is an emerging resistance to permethrin in different regions of the world [1,5,8,9,10,11]. Permethrin resistance in head lice is a complicated process and involves the development of mutations related to knockdown resistance (kdr). One of the target sites for permethrin is the voltage-sensitive sodium channel (VSSC) gene [9,10,11,12]. Kdr mutations in this gene are responsible for encoding the sodium channel and cause alterations to the structure of the sodium channel protein [10,11,12,13,14]. Previous studies on head lice have revealed the emergence of single nucleotide polymorphism (SNPs) associated with knockdown resistance (kdr) to permethrin on M815I (ATG > ATT), T917I (ACA > ATA), and L920F (CTT > TTT) regions of voltage-sensitive sodium channel (vssc) genes, where mutations in M815I and L920F are associated with reduced susceptibility to permethrin [12,13,14,15,16,17,18]. The T917I mutation, whether on its own or in combination with the M815I or L920F mutations, is significant in conferring resistance to permethrin and can serve as a molecular marker for the resistance of head lice to permethrin and pyrethroids [17,18]. These alterations are all located on the IIS1-2 extracellular loop of the α-subunit of the VSSC gene and could eventually develop resistance to permethrin by lowering permethrin’s binding and blocking capacities in VSSC, thus making it safe for the lice [12,15,18,19,20]. In addition, Gao, et al. have demonstrated that the resistance traits were completely recessive [21]. Hence, it is essential to identify these mutations with suitable methods to unveil the severity of permethrin resistance in head lice treatment.

Although there are many reports on the identification of gene mutations associated with permethrin resistance in head lice from different regions of the world, there is yet only one report from Türkiye [9] and none from Nepal, to our knowledge. Thus, in the present study, we aimed to assess the permethrin-resistance-related gene mutations in head lice samples collected initially during field studies on primary school children in two cities of Türkiye and Nepal by using molecular methods.

## 2. Materials and Methods

**Study Group:** This study was performed using the data collected previously in two pediatric research projects conducted in Istanbul and Nagarkot provinces of Türkiye and Nepal, respectively. [3,22].

**Figure 1:**
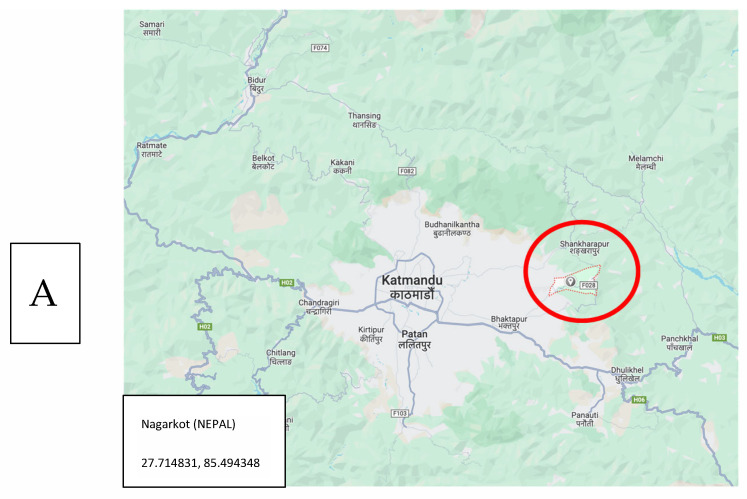

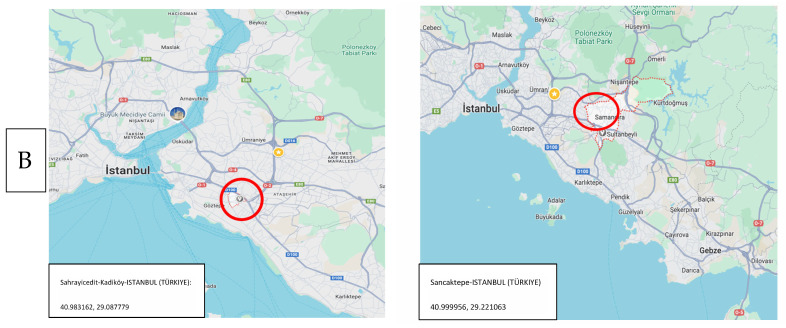
Locations of the study sites in Nagarkot (Nepal; (**A**)) and Istanbul (Türkiye; (**B**)).

**The Istanbul Project:** Two primary schools, one in the Sahrayicedit district of Kadiköy county, and one in Sancaktepe county (Figure 1) located on the Asian side of Istanbul, were selected for this study upon the previous application of the school directors to the Local Office of the Ministry of Education for the detection and treatment of HLI-positive children within the academic years of 2014 and 2015. After receiving consent from the Ministry, followed by that of the parents and children, a total of eight school visits were scheduled in coordination with the school directors for the examination of the children’s hair and scalp. The project was conducted on a total of 340 primary school children aged between 6 and 12 years, and a total of 101 head lice were collected from 32 children with HLI (5 boys and 27 girls). The prescheduled school visits were conducted by four medical students of Acibadem University, in coordination with a teacher and a high school student. The enrolled children were examined using special combs, named “PDC (Precision Detection Comb, KSL Consulting, Copenhagen-DENMARK)”, which were specially designed for both the detection and removal of head lice. The combing of the hair of the school children was carried out as described previously [1]; moreover, special emphasis was given to combing the hair of the girls, since almost all of them had long hair, and initial soaking of the hair was necessary for effective combing with PDC. The hair of each child was combed from forehead to neck at least three times during the examination, while the removal of all adult lice was aimed at all of the time and maintained during the examinations. The collected lice were put immediately into vials containing 96% ethanol, and they were kept in the deep freezer (−20 °C) in the laboratory until the day of the molecular assessments.

**Field Studies in Nepal:** Assessments on HLI were conducted in rural regions of Nagarkot city of Nepal (Figure 1) in July 2015 during the execution of the Social Pediatrics Project by volunteer students and academicians of Acibadem University, in collaboration with the local authorities and a Turkish non-governmental organization (MEDAK: Medical Rescue Association) [22]. A total of 159 children aged 5–12 years were combed using PDC, from forehead to neck at least three times, while the removal of all adult lice was aimed at and maintained during the examinations. A total of 99 head lice were identified from 26 children (16.4%) during the examinations and put immediately into vials containing 96% ethanol. They were kept in the deep freezer (−20 °C) in the laboratory until the day of the molecular assessments.

**Molecular Assessments:** A total of 96 Turkish and 96 Nepalese head lice samples were assessed using PCR. Initially, the total genomic DNA of the lice was extracted using a commercial kit (GMBiolab Tissue & Cell Genomic DNA Purification Kit (GenemarkBio), Taipei, Taiwan), according to the manufacturer’s suggestions. DNA concentration and purity were assessed using the MaestroNano (Maestrogen, Bangkok, Thailand) nanodrop device. The primers, *pedic815F2* (5′-GGCCTTACTTGTATTCGACCC-3′), *pedic815R2* (5′-CCCAAAGCTTCAACAGTTTG-3′), and kdr-F (5-AAATCGTGGCCAACGTTAAA-3), which covered the knockdown resistance (kdr)-related M815I (ATG > ATT) mutation site on the VSSC gene (GenBank ID: OM891554.1) of *P. h. capitis*, were designed initially. In addition, the primers kdr-F (5-AAATCGTGGCCAACGTTAAA-3) and kdr-R (5-TGAATCCATTCACCGCATAA-3) covering the knockdown resistance (kdr)-related T917I (ACA > ATA) and L920F (CTT > TTT) mutation sites on the VSSC gene (GenBank ID: OM891554.1) of *P. h. capitis* were also designed, as described [19]. These primer pairs were for used separately for PCR amplifications. PCR tests were performed with a total of 50 μL standard reaction volume for each sample. Optimum amplification conditions were obtained with 50-ng genomic DNA, 1× reaction buffer, 2.5 mM MgCl_2_, 20 μM dNTPs, 0.3 μM primer, and 1U Taq DNA polymerase (GMbiolab, Taipei, Taiwan), and a PCR mix was prepared. The conditions of the PCR tests comprised an initial denaturation step of 5 min at 95 °C, followed by 35 cycles at 95 °C for 45 s (denaturation), 57 °C for 45 s (annealing), and 72 °C for 60 s (extension), and lastly with a final extension period of 5 min at 72 °C. A PCR mix without any template DNA was used as the negative control to test for any contamination. The PCR products were sequenced with the “BigDye cycle sequencing kit” (Applied Biosystems, Foster City, CA, USA) using an ABI 3130 XL genetic analyzer (Applied Biosystems). The voltage-sensitive sodium channel α-subunit gene sequence was aligned and analyzed with MEGA 7^®^ software. The Hardy–Weinberg equilibrium (HWE), linkage disequilibrium (LD), and haplotype analysis were all used in the assessments using SHEsis software (https://www.nature.com/articles/7290272 Access Date: 4 December 2024).

## 3. Results

**Figure 2:**
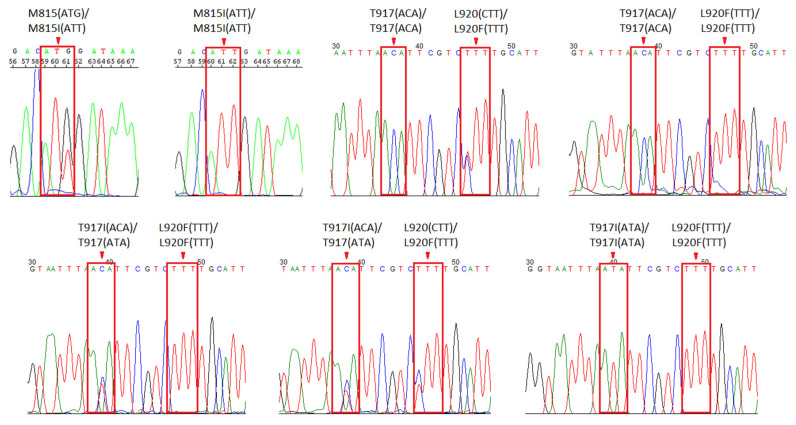
Chromatograms of kdr-like M815I (ATG > ATT), kdr T917I (ACA > ATA), and kdr-like L920F (CTT > TTT) mutation sites are presented. Chromatograms of the PCR products demonstrate kdr-related nucleotide signals on M815I (ATG > ATT), T917I (ACA > ATA), and L920F (CTT > TTT) mutation sites.

A total of 192 DNA samples (96 each) were amplified by PCR, and the permethrin-resistance-related gene regions were assessed by Sanger sequencing. The results showed that at least one of these three mutations, M815I (ATG > ATT), T917I (ACA > ATA), or L920F (CTT > TTT), were detected on the VSSC gene of *P. h. capitis* in the lice samples from both Türkiye and Nepal (Figure 2). However, the rates and co-occurrence of these mutations showed significant differences between them (Table 1). The genotypic distribution of the M815, T917, and L920 regions in the lice samples from Türkiye were consistent with HWE (*p* > 0.05). However, regarding the lice samples from Nepal, consistency with HWE was present in both the T917 and L920 regions (*p* > 0.05) but was absent from the M815 region. In addition, while the alleles associated with kdr-related M815I (ATG > ATT), T917I (ACA > ATA), or L920F (CTT > TTT) mutations on the VSSC genes were all identified in the lice samples from Türkiye, alleles associated with the kdr-related T917I (ACA > ATA) mutation were found to be absent in all tested lice samples from Nepal (Table 2).

**Figure 3:**
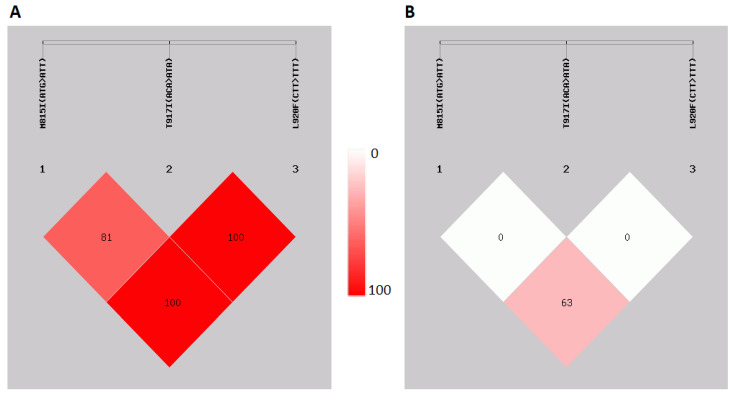
Linkage disequilibrium test of the three (M815I (ATG > ATT), T917I (ACA > ATA), and L920F (CTT > TTT)) mutation sites of the lice samples from Türkiye (**A**) and Nepal (**B**). The color bar indicates the power of the LD analysis results.

The linkage disequilibrium test and haplotype analysis were applied to three mutation sites, M815I (ATG > ATT), T917I (ACA > ATA), and L920F (CTT > TTT), during this study. The results have revealed a strong linkage disequilibrium between these three mutation sites in the Turkish samples (Figure 3A). However, only a moderate level of linkage disequilibrium was identified between the M815I and L920F (D’ = 0.630) sites in the lice samples from Nepal (Figure 3B).

The co-occurrence rates of both wild-type and mutated nucleic acids associated with three different mutation regions on the VSSC gene of the head lice samples from Türkiye and Nepal (M815I (ATG > ATT), T917I (ACA > ATA), and L920F (CTT > TTT)) are demonstrated in Table 3. A total of six possible haplotypes are listed, and the leading haplotype in the lice samples from Türkiye was found to be the “TTT” haplotype (91.7%). This means that 91.7% of the lice samples from Türkiye tested in this study have alleles associated with those three kdr-related mutations on the VSSC gene. In addition, the “GCC” haplotype, which is known to lack these mutations, was found to be 0% in Türkiye. In Nepal, the leading haplotype was the “TCT” haplotype, with a rate of 48.1%, and the “GCC” haplotype, which does not have these mutations in Nepal, was found to be 33.0%.

## 4. Discussion

The primary option to control HLI in patients has long been the topical application of pediculicides, such as permethrin, on the scalp intensively. However, there is a growing pile of assessment results that indicate both the toxic outcomes of permethrin use in children and an emerging resistance to permethrin and other pyrethroids, lately [2,3,4,15,16,17,18,19,20,21]. The existing literature on permethrin resistance in head lice presents a comprehensive overview of the global landscape, revealing both commonalities and distinct regional variations in resistance patterns. Numerous studies [2,3,4,5,6,7,8,9,10,11,12,13,14,15,16,17,18,19,20,21,22,23,24,25,26,27,28] indicate a recurrent pattern that is associated with specific mutations, such as T917I, L920F, and M815I, as significant contributors not only to permethrin resistance, but also to the development of kdr in head lice populations. The theory indicating that the mutations M815I and L920F occur alongside the mutation T917I has resulted in the identification of two hotspot regions on the VSSC gene of head lice. These include the M815, T917, and L920 sites of the gene and are used to indicate the frequency of kdr resistance alleles in head lice populations [12,13,17]. Contrary to previous studies, the present study has revealed, for the first time, that the M815I and L920F mutations could occur together on VSSC, as observed in the lice samples from Nagarkot, Nepal. In other words, the T917I mutation, which was common in the lice samples from Türkiye, was detected in none of the samples from Nepal. Notably, these mutations exhibit persistence across diverse geographic locations and populations [9,11]. Molecular trials conducted on *P. h. capitis* De Geer, 1767, from different research centers unveil the high frequency of these mutations on the VSSC gene, consistently, and thus underline their fundamental role in conferring resistance to permethrin [13,17,25,26]. This unified genetic profile suggests a shared molecular basis for pyrethroid resistance in head lice and highlights the important roles of these specific mutations in both the development and monitorization of resistance.

On the other hand, distinct patterns of emerging resistance may be noticed during the examination of regional variations. In a study from Buenos Aires, Argentina, a pervasive resistance with highly resistant head lice populations was reported [27]. Conversely, in a study from Saudi Arabia, a relatively lower frequency of resistance was identified, which suggests an intriguing regional dichotomy in resistance levels [28]. Moreover, the resistance mechanisms elucidated in different regions exhibit remarkable diversity. In a study from Israel, a multifaceted resistance profile involving both GST-based resistance and weak monooxygenase mechanisms was presented [29]. However, two studies from Iran and Denmark pinpointed head-lice-population-specific findings and the target site insensitivity as the predominant resistance mechanisms in head lice [26,30]. In the only previous study from Türkiye, the identification of different clades (A and B) of head lice introduced a unique population structure [9]. In Madagascar, the intersection of resistance and bacterial pathogens in head lice was found to expand the discourse and has underlined the need for a holistic approach to understand HLI epidemiology [31]. This integrated perspective opens a path for more comprehensive management of HLI in the public, involving the consideration of both genetic and environmental factors. These studies collectively underscore the importance of ongoing methodological innovations, global collaboration, and regional analyses to support management strategies.

In the present study, we used head lice samples collected from primary school children in Istanbul, Türkiye, and Nagarkot, Nepal, to assess the gene mutations associated with permethrin resistance for the first time. The overall frequency (%) of the M815I, T917I, and L920F kdr mutations was found to be relatively higher in the Turkish samples compared to the Nepalese samples (96.9, 92.2, and 99.0 for the Turkish samples; and 56.8, 0.0, and 58.3 for the Nepalese samples, respectively). Our assessments clearly demonstrated that, compared to the Turkish samples, and the figures reported in previous articles from Iran, Canada, and the US, the levels of kdr mutation in the Nepalese head lice samples were relatively more modest [20,21,23]. This is probably due to the limited use of permethrin in Nepal for HLI, especially in rural regions that are remote from regular health services and have limited financial resources, as mentioned in epidemiological studies [2,7,15,19]. In Türkiye, high rates of HLI have been reported in primary school children from all parts of the country, while ineffective applications of permethrin for HLI have been more pronounced in recent years [3,5,9,24]. In one of our previous studies conducted on primary school children in Istanbul (who were attending in one of two schools selected for the present study), we examined the live effects of permethrin in comparison with a new anti-pediculicidal agent using a stereomicroscope and observed that 43 of 72 (59.7%) lice in this study remained alive despite just being exposed to permethrin, even after 24 h (unpublished data). In addition, it was recently reported that the sales figures of new pediculicides, such as dimethicone, have doubled in Türkiye between 2015 and 2022, while there was a remarkable decline in the sales figures of permethrin, despite its lower price. This decline may be associated with the observed lowering treatment efficacy of permethrin in Türkiye lately, which may be the result of the emerging resistance [3,5,24].

The HWE test results of the lice samples from Istanbul and Nagarkot revealed some interesting results as well. This test was used to score the genotype frequencies of the lice populations, and it was found that the observed kdr-like M815I (0.000620) and kdr T917I (0.000000) carrier lice genotype frequency was very close to the expected rates of the entire Istanbul sample. In contrast, the test revealed that the observed kdr-like M815I mutation rate differed from the expected rates of the Nagarkot samples. However, the test revealed that the lice belonging to the Nagarkot lice population were not of the mutant kdr T917I genotype, but of the wild-type T917 (0.001552) genotype, which was close to the expected population. In addition, according to the linkage disequilibrium test, the three kdr-related loci, M815I, T917I, and L920F, were co-inherited strongly among the Istanbul samples. However, the results of the linkage disequilibrium test were in contrast with the results of the Nagarkot samples in lower LD power results. Another finding of this study was that high LD power results indicated the presence of excess homozygotes among the lice population of Istanbul, which was associated with inbreeding or infixation of mutant M815I, T917I, and L920F alleles. This pattern was similar to those reported in the lice populations of Iran, Canada, and USA previously [20,21,23]. Conversely, the Nagarkot lice population displayed a low level of LD power, which indicates that the majority of the Nepalese head lice had an abundance of kdr-type mutation heterozygotes and displayed the presence of active selective pressure on Nagarkot’s head lice population. This pattern was observed in Thailand, another country with relatively lower rates of permethrin use [15].

The outcomes presented in this study indicate the presence of mutations associated with permethrin resistance in head lice samples collected in Istanbul, the largest province of Türkiye, for the first time. They are concordant with the only previous study from Türkiye, where high resistance allele frequency was detected in three mutation sites (T917I, L920F, and M815I) of 150 head lice samples from Manisa province, 400 km south of Istanbul [9]. In addition, a decline in the sales figures of permethrin between 2015 and 2022 [24], and our previous unpublished study where we observed high levels of live head lice under a stereomicroscope, even 24 h after exposure to permethrin, contribute to permethrin resistance in head lice in Türkiye. However, the rates of the mutations associated with permethrin resistance in head lice from Nepal was relatively lower in our study. This is probably due to the lower rates of permethrin use, especially in the study site in Nagarkot. There are limited published studies on the epidemiology of HLI in Nepal [6,32], even though it can also be a nuisance for school children there, as well. In addition, it has been shown that head lice harbor some life-threatening bacteria, such as *B. quintana* and *Acinetobacter* spp., and are potential vectors of them for humans, despite the absence of proof of transmission at the moment [33,34,35,36].

There are important limitations of the present study. Since it was not initially planned or retrospectively applied to DNA samples of the collected head lice samples from previous field studies in Istanbul and Nepal, where no published data were available on permethrin resistance nor any related mutations before, the requirements of this planned study were not fulfilled, due to a limited budget. These requirements primarily include the biochemical testing of the head lice samples and the assessment of the presence of active permethrin resistance in lice, which was impossible as the study samples were of DNA. However, the addition of the information of our unpublished data, which were gathered in our laboratory in Istanbul with live lice collected from the primary school in Sahrayıcedit District of Kadiköy county (one of the study sites of the present study) revealed active resistance in almost 60% of the tested lice, which is an important point to mention. Proteomic analyses may also be carried out in future studies with adequate funds.

## 5. Conclusions

The results of this first study in Istanbul (Türkiye) and Nagarkot (Nepal) indicate that almost all tested head lice in Istanbul and more than half in Nagarkot have gene mutations associated with permethrin resistance. These mutations are very common in Türkiye, since permethrin has been a cheap and well-known pediculicidal agent there for decades; however, they are less common in head lice in Nepal, probably due to permethrin’s insufficient delivery to patients in Nepal, especially in remote areas of the country, due to economical and geographical constraints. Despite being more expensive, new pediculicidal agents, such as dimethicone, offer safer and more effective treatment today, and thus will gradually replace permethrin for HLI treatment, as expected.

## Figures and Tables

**Table 1:** Distribution of the tested head lice samples from Istanbul (Türkiye, (n = 96)) and Nagarkot (Nepal, (n = 96)), according to their genotypes.

	Genotype	Samples (n)	Frequency (%)	Total (%)	H–W (χ^2^) ^a^	Pearson’s *p* ^b^
Türkiye	Homozygous M815I/M815I	93	96.9	100		
	Heterozygous M815I/M815	0	0		0.000620	0.980118
	Homozygous M815/M815	3	3.1			
Nepal	Homozygous M815I/M815I	31	32.3	100		
	Heterozygous M815I/M815	47	49		96.00	1.23 × 10^−22^
	Homozygous M815/M815	18	18.7			
Türkiye	Homozygous T917I/T917I	83	86.4	100		
	Heterozygous T917I/T917	11	11.5		0.000000	1.000000
	Homozygous T917/T917	2	2.1			
Nepal	Homozygous T917I/T917I	0	0	100		
	Heterozygous T917I/T917	0	0		0.001552	0.085101
	Homozygous T917/T917	96	100			
Türkiye	Homozygous L920F/L920F	94	97.9	100		
	Heterozygous L920F/L920	2	2.1		2.015510	0.095143
	Homozygous L920/L920	0	0			
Nepal	Homozygous L920F/L920F	38	39.6	100		
	Heterozygous L920F/L920	36	37.5		0.010637	0.917834
	Homozygous L920/L920	22	22.9			

^a^ Populations were tested for Hardy–Weinberg equilibrium (HWE), using a Chi-square test. Genotypic distribution is considered consistent between the observed and expected population when HWE was found to be below 3.84 (df = 1). ^b^ Pearson’s probability (*p*) values of the Chi-square test.

**Table 2:** Distribution of the alleles associated with kdr-like mutations on M815I (ATG > ATT), T917I (ACA > ATA), and L920F (CTT > TTT) mutations of the VSSC genes in lice samples from Türkiye (n = 96) and Nepal (n = 96).

	Allele	Frequency (%)	95% Confidence Interval
Türkiye	M815I (Permethrin-resistant)	96.9	96.1–97.7
	M815 (Permethrin-susceptible)	3.1	2.3–3.9
Nepal	M815I (Permethrin-resistant)	56.8	53.3–60.5
	M815 (Permethrin-susceptible)	43.2	39.7–46.7
Türkiye	T917I (Permethrin-resistant)	92.2	90.8–93.6
	T917 (Permethrin-susceptible)	7.8	6.4–9.2
Nepal	T917I (Permethrin-resistant)	0	-
	T917 (Permethrin-susceptible)	100	-
Türkiye	L920F (Permethrin-resistant)	99.0	98.7–99.3
	L920 (Permethrin-susceptible)	1.0	0.7–1.3
Nepal	L920F (Permethrin-resistant)	58.3	53.2–63.4
	L920 (Permethrin-susceptible)	41.7	36.6–46.8

**Table 3:** Haplotype analysis of the three (M815I (ATG > ATT), T917I (ACA > ATA), and L920F (CTT > TTT)) mutation sites of the lice samples from Türkiye (A) and Nepal (B) *.

Haplotype	Head Lice (*Pediculus humanus capitis*) Samples (Frequency %)	
	Türkiye	Nepal
G C T	5.00 (0.026)	19.57 (0.102)
G T T	1.00 (0.005)	0.00 (0.000)
T C C	2.00 (0.010)	16.57 (0.086)
T C T	8.00 (0.042)	92.42 (0.481)
T T T	176.00 (0.917)	0.00 (0.000)
G C C	0.00 (0.000)	63.43 (0.330)

* The haplotypes with a frequency of <0.03 were ignored in analysis.

## Data Availability

The original contributions presented in this study are included in the article. Further inquiries can be directed to the corresponding author.
